# The Use of Hydrogels in the Treatment of Metal Cultural Heritage Objects

**DOI:** 10.3390/gels9030191

**Published:** 2023-03-02

**Authors:** Elodie Guilminot

**Affiliations:** Arc’Antique Conservation and Research Laboratory, 26 Rue de la Haute Forêt, 44300 Nantes, France; elodie.guilminot@loire-atlantique.fr

**Keywords:** hydrogel, agar, conservation-restoration, cleaning treatment, stabilization, iron, copper alloys, lead, Al alloys, silver

## Abstract

Currently gels are widely used in the restoration of paintings, graphic arts, stuccowork and stonework, but their use in metal restoration is less widespread. In this study, several polysaccharide-based hydrogels (agar, gellan and xanthan gum) were selected for use in metal treatments. The use of hydrogels allows to localize a chemical or electrochemical treatment. This paper presents several examples of treatment of metal objects of cultural heritage, i.e., historical or archaeological objects. The advantages, disadvantages and limits of hydrogel treatments are discussed. The best results are obtained for the cleaning of copper alloys via associating an agar gel with a chelating agent (EDTA (ethylenediaminetetraacetic acid) or TAC (tri-ammonium citrate)). The hot application allows to obtain a peelable gel, particularly adapted for historical objects. Electrochemical treatments using hydrogels have been successful for the cleaning of silver and for the dechlorination of ferrous or copper alloys. The use of hydrogels for the cleaning of painted aluminum alloys is possible but it has to be coupled with mechanical cleaning. However, for the cleaning of archaeological lead, the cleaning using hydrogels was not very effective. This paper shows the new possibilities of using hydrogels for the treatment of metal cultural heritage objects: agar is the most promising hydrogel.

## 1. Introduction

When a metal object is discarded, the metal tends to revert to its natural mineral state and corrosion takes place. The resulting corrosion products form layers that vary considerably depending on the metal and its exposure to environment: iron corrosion products are often voluminous and can be highly reactive, especially in the presence of chlorides, while Pb objects tend to form a highly protective, thin corrosion layer, except when organic acids are present. A conservators’ objective is to locate the original surface of the object, which is often obscured by the various layers of corrosion. 

Work carried out by conservators must be the least invasive possible while ensuring the stability of the object and restoring its legibility. Ideally, a cleaning treatment would act on a single element (generally corrosion products, sediments or dust) without affecting the layer(s) to be preserved. A range of stabilization and cleaning techniques has been developed and their effectiveness proven: these techniques may be mechanical, chemical or electrochemical [1]. The most common mechanical cleaning methods involve removing corrosion products with a scalpel, brush or compressed air, or by sandblasting. These mechanical techniques are widely used but can be very time consuming and are unsuitable for some objects. Chemical cleaning involves the use of chelating agents or acids to selectively remove corrosion products. Electrochemical treatments are used to remove chlorides or reduce corrosion products. However, chemical and electrochemical treatments require the object to be immersed in the treatment solution. If the object is composed of more than one material, the use of this type of treatment is largely ruled out. Moreover, if chemical solutions penetrate too far into the object, this can result in a weakening of the layers underlying the original surface. 

Conservators often have to combine different techniques to obtain a satisfactory result and are always looking for new protocols that allow them better control. For this reason, the Arc’Antique laboratory has been testing and developing gel-applied treatments for metals for several years. In the early 1990s, Wolbers came up with an innovative methodology to treat paintings: the application of liquid cleaning agents embedded in gel matrices [2,3]. In conservation, the term “gels” is often linked with other words, such as packs, pastes, poultices, compresses and pads, to indicate that localized cleaning is intended. The gel acts as a vehicle for applying the treatment solution to the surface to be cleaned. Gelled formulations are used to lengthen solution retention time and to improve control over the cleaning process. Many formulations are common in conservation–restoration [4,5]: polyacrylic (Pemulen^®^ or Carbopol^®^), cellulose-based (CMC (carboxymethylcellulose) and cellulose ether), polysaccharides (agar, gellan gum or xanthan gum), emulsions, and poly(vinyl alcohol) (PVA-borax and gels developed by the University of Florence (Center for Colloid and Surface Science: CSGI)).

Interactions between polymer chains create a 3D structural network in gels. These interactions can be in the form of a hydrophobic, electrostatic or van-der-Waals interactions or hydrogen bonds: the system is then called a “physical gel” [6]. If connections between different polymer chains are due to covalent bonds, the gel is known as a “chemical gel”. Gels can also be classified according to the nature of the solution embedded in the gel: In the case of a liquid phase composed of an organic solvent, they are referred to as organogels; if the solvent is water, they are called hydrogels. For the restoration of cultural heritage metal, physical hydrogels are preferred for various reasons: low cost, accessibility, ease of processing and good compatibility with the active agents most commonly used by restorers.

In this article, the study will focus on physical hydrogels based on polysaccharides: agar, gellan gum and xanthan gum [7,8]. Normally, physical gels contain reversible bonds formed by temporary associations between chains. These associations have finite lifespans and are continuously breaking up and reforming. These weak physical bonds are often hydrogen bonds, but can also be a formation of block copolymer micelles and ionic associations [9]. However, some physical hydrogels form more stable bonds. The strong physical bonds between polymer chains are effectively permanent within a given set of experimental conditions. Examples of strong physical bonds are lamellar microcrystals, glassy nodules, and double and triple helices. Hence, these physical hydrogels are analogous to chemical gels. Agar can form a semi-rigid network. It is composed of a mixture of agarose and agaropectin in variable proportions depending on the type of algae used and the manufacturing process [10]. The linear chains of agarose arrange themselves into double helix structures that aggregate to form “suprafibers” comprising anywhere up to 10^4^ helices. Agar gels have the remarkable property of reversibility. They simply melt on heating and solidify again upon cooling. These transformations can be repeated indefinitely in the absence of aggressive substances. Generally, 1–5% wt agar is mixed in aqueous solution and heated up to 85 °C. It then develops a random coil structure which has the ability to progressively rearrange itself and gel during the cooling stage, at below 40 °C [11,12]. The gelling temperature of agar is 38–42 °C. So, when the hot agar gel is cooled on a surface, the film adheres to the surface and is easy to remove. These gels are called peelable gels [13]. It was shown that the agar concentration strongly influences the water state within the gel network: agar at 1% wt releases far more water than gels at 3% and 5% wt, acting as a “free water reservoir” [14]. Conservators can adapt the rigidity of the gel film to suit the surface to be treated and adapt its sensitivity to water.

The use of agar gels in the cleaning of artworks has been studied for more than a decade and its application concerns a variety of substrates with different physico–mechanical features: from stone [15,16] and plaster surfaces [17] with compact soiling, to the most fragile, such as paper [18,19] or paintings [11,20,21]. Several research studies have recently been launched with the aim of understanding the cleaning mechanism of agar and optimizing its use in the restoration of heritage building materials [13,14,16,17,22]. These studies have shown the possibility of successfully using chelating agents combined with agar gels to remove corrosion product stains from marble.

Gellan gum has similar characteristics to a peelable gel. It is a linear, anionic heteropolysaccharide produced by a microorganism (Sphingomonas elodea) [23]. The molecules are transformed into an ordered double-helical conformation upon cooling, followed by associations between the helices through weak interactions, such as hydrogen bonds and van-der-Waals forces. In the presence of cations (especially Ca^2+^), gellan gum forms hard, brittle gels that are able to promote and stabilize the ordered “crystalline-like” structure. However, the addition of cations is not necessary for the formation of high-acyl gellan gum gels [24], that have a higher gelling temperature than agar gels: about 70 °C. Another difference with agar is that gellan gum is less thermo-reversible: once the film is formed, conservators cannot reuse it (or any excess gel prepared). Gellan gum is mainly used to clean paper because it keeps water damage to a minimum, and thus more effectively preserves the integrity of the original, historical paper [25,26]. Sometimes gellan gum is not used as a rigid peelable gel, it can be prepared cold and form a viscous gel. For example, if biopatin is applied, the integration of microorganisms requires that the gel must not be heated during the preparation process [27].

Unlike agar or gellan gum, xanthan gum has two conformations: an ordered conformation (helix) and a disordered one (random coil) [28]. This double conformation excludes the possibility of obtaining a peelable gel, as is possible with agar or gellan gum gels. Xanthan gum is an extracellular polysaccharide composed of glucose and secreted by Xanthomonas Campestris bacteria. Xanthan gels obtained using dissolution at ambient temperatures tend to be highly viscous. While the stability of peelable gels (agar or gellan gum) is strongly pH-dependent, as the double helix structure is maintained in neutral pH, xanthan gels maintain a high viscosity in a wide pH range [29]. The main disadvantage of these viscous gels is residue [30].

In this paper, different physical hydrogels based on polysaccharides (agar, gellan gum and xanthan gum) were tested for the cleaning and stabilization of metal cultural heritage objects. In each case study, the main research issue is presented by detailing the objectives of the restoration, the cleaning protocols tested and the relevant results.

## 2. Results

### 2.1. Choice of Treatments

Choice of treatment was determined by the selection of the active agent needed to clean or stabilize the object. Conservators know which active agents are suitable for cleaning relevant metals in chemical treatments. Generally, to remove copper corrosion products, solutions with chelating agents are used, such as EDTA (ethylenediaminetetraacetic acid) or TAC (tri-ammonium citrate). In the case of lead corrosion products (based on lead carbonates), acidic solutions are also recommended. For cleaning silver, electrolytic reduction yields good results. Electrolysis is also used for the stabilization of ferrous or copper objects.

The use of gels allows the quantity of active agents to be reduced and their degree of penetration into the object limited. A gel treatment can also be used to treat a surface locally, enabling the whole object to be preserved. Previous studies have shown the compatibility of different gels with the active agents most widely used by conservators [7,31]. Physical hydrogels based on polysaccharides with a helix structure (agar and gellan gum) remain stable in a neutral pH range (about 5 to 8). If the treatment solutions are more acidic or basic, it is possible to form gel films with demineralized water and then immerse the film in the treatment solution for 2 h. The gel film maintains its mechanical strength throughout the application (treatment time of less than 2 h); however, contact with the object’s surface is less effective than when the gel is applied hot. Xanthan gels do not form rigid gels and can be used in a wider pH range (between 4 and 10).

Table 1 summarizes the compatibility between treatment solutions and polysaccharide-based hydrogels (agar, gellan gum and xanthan gum).

### 2.2. Case Study

#### 2.2.1. Cleaning of Copper Alloys—Historical Artifact

**Figure 1:**
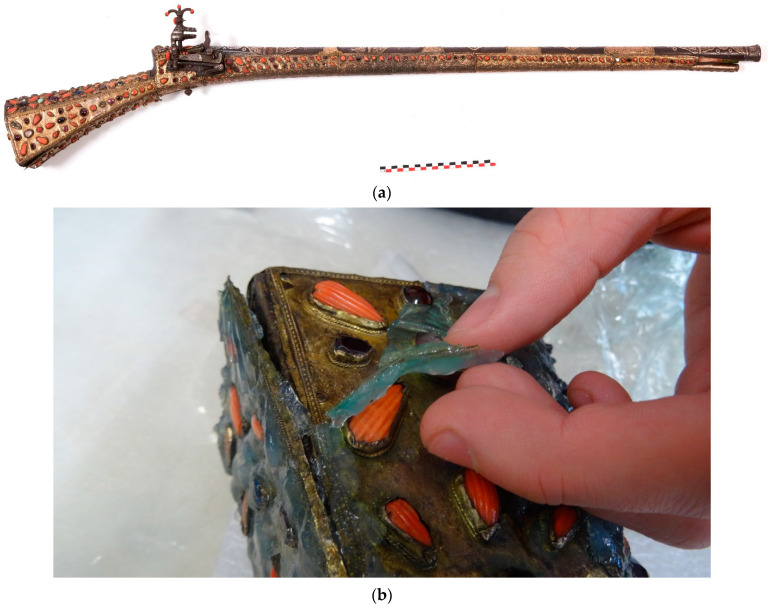
The gun from the Pierre Loti Museum collection (**a**), removal of the agar gel (**b**) (© J.G. Aubert/Arc’Antique—Grand Patrimoine de Loire Atlantique, Nantes, France).

Our first case study concerns the cleaning of objects from the Islamic art collection of the French author, Pierre Loti, whose former home is now a French museum. Located in Rochefort, the author’s collection of art objects is remarkable for its scope and features a wealth of various typologies, creative techniques and materials. The case of the gun (Figure 1) illustrates the problems involved in cleaning a tarnished copper alloy. The object also typifies the complexity of treating composite objects because it is constituted of a wooden butt covered with copper alloy plates decorated with semi-precious gems and corals. The copper alloy plates displayed uniform, light corrosion, and were stained with grease deposits. The multi-material nature of the object made chemical treatment by immersion impossible. Mechanical cleaning was possible but would have been long and difficult because of the decoration. Gel cleaning provided an attractive alternative. After initial cleaning with a mixture of water/ethanol applied using a cotton swab, the surface was de-greased with acetone. The different gels (agar, gellan gum and xanthan gum) were tested with a TAC solution at 2.5 wt% on the copper alloy plates. The best results were achieved using agar gel applied hot. Application time can vary between 10 min and 1 h, depending on the conservator’s cleaning objective. Gel treatment successfully removed most of the copper corrosion products. The surface was rinsed with demineralized water applied with a cotton swab. The cleaning resulted in a homogeneous surface, as shine could be controlled. 

Gellan gum gels also gave good results but processing was more delicate. The gel rapidly rigidified because of its higher gelation temperature than agar. Xanthan gum gels also removed corrosion products but left a lot of residues on the surface.

These treatments were also carried out by applying the gel as a film: Agar gel films were pre-made with demineralized water and immersed in a TAC solution before application. The cleaning was effective due to good contact between the gel film and the object. However, if an air bubble forms between the gel film and the object, the area remains tarnished.

#### 2.2.2. Cleaning of Gilded Copper Alloys—Historical Artifact

**Figure 2:**
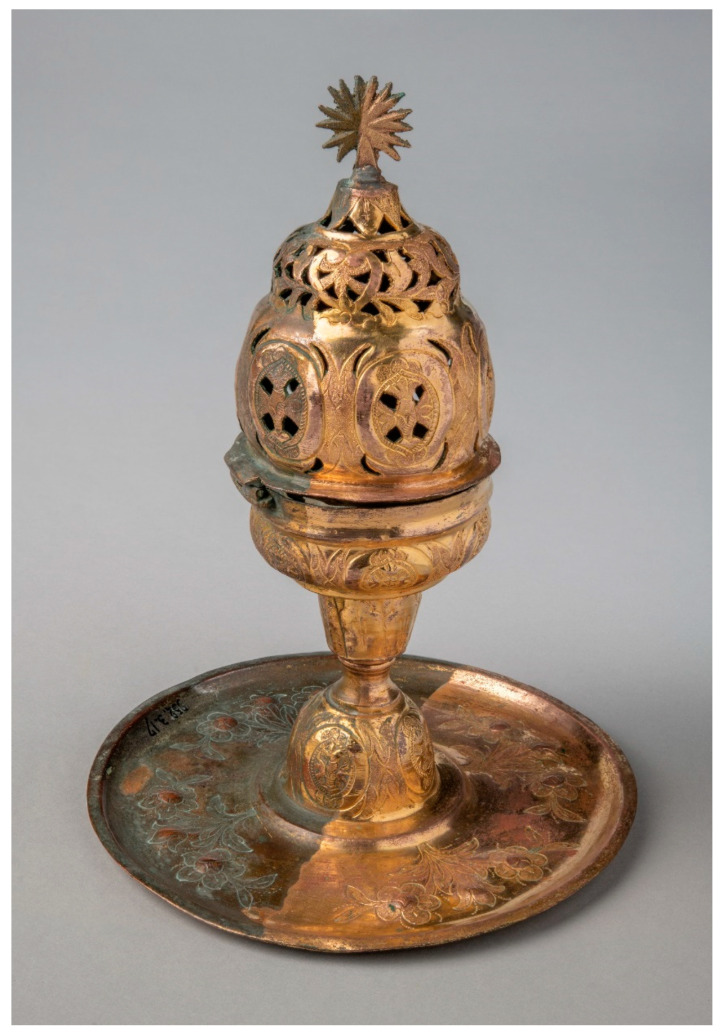
The Armenian censer from the Dobrée Museum collection during treatment (© L. Preud’homme/Arc’Antique—Grand Patrimoine de Loire Atlantique, Nantes, France).

The second case study also concerns a historical object: an Armenian censer from the Dobrée Museum collection (Figure 2). The object is made of gilded copper with a set of chased, embossed and openwork decorations. The copper showed thick corrosion masking the gilding (sometimes with gaps). The surface of the object was also covered with a thick, dirty varnish. An application of agar gel with tetrasodium EDTA (5 wt%) for 5 min removed dirt, varnish and part of the corrosion products. Cleaning was completed by the application of an agar gel with di-EDTA (2.5 wt%) for 5 min. Then, the surface was rinsed with demineralized water using a cotton swab. Agar gel cleaning produced a result that conservators consider to be highly satisfactory.

#### 2.2.3. Cleaning of Copper Alloys—Archaeological Artifact

**Figure 3:**
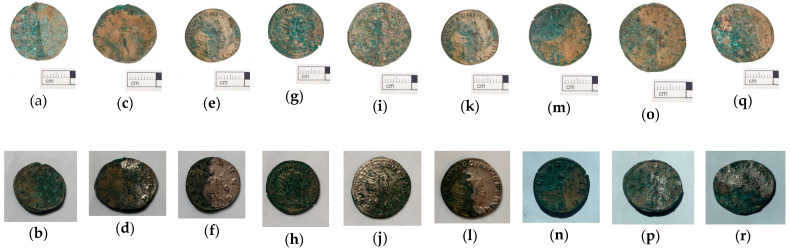
Coins from the Cléons treasure (only half of the coin’s surface was treated in each case) before or after treatments: before (**a**) and after 5 applications of agar with di-EDTA 0.5 wt% (**b**), before (**c**) and after 5 applications of agar with di-EDTA 2 wt% (**d**), before (**e**) and after 5 applications of agar with di-EDTA 5 wt% (**f**), before (**g**) and after 5 applications of gellan with di-EDTA 0.5 wt% (**h**), before (**i**) and after 5 applications of gellan with di-EDTA 2 wt% (**j**), before (**k**) and after 4 applications of gellan with di-EDTA 5 wt% (**l**), before (**m**) and after 5 applications of xanthan with di-EDTA 0.5 wt% (**n**), before (**o**) and after 5 applications of xanthan with di-EDTA 2 wt% (**p**), before (**q**) and after 5 applications of xanthan with di-EDTA 5 wt% (**r**) (© C. Colonnier/Arc’Antique—Grand Patrimoine de Loire Atlantique, Nantes, France).

The first two case studies showed that gel cleanings are effective in removing corrosion from copper. The third case study presents a similar problem: the removal of copper corrosion products from silver-plated copper alloy coins. In this case, however, the coins were archaeological objects, from the Cléons treasure (Haute Goulaine, Pays de Loire, France) dating from Antiquity (between 271 and 274 CE). They are in the effigy of various Roman emperors: Volusianus, Valerianus I, Gallienus and Saloninus. The Cléons treasure was discovered in 1901 and is conserved in the Dobrée museum collection. These coins were used in a study comparing cleaning with a range of gels, published by Giraud et al. [7]. In this article, we will focus on the tests performed with hydrogels: agar, gellan gum and xanthan gum (Figure 3). The objective of the cleaning was to reveal the significant surface which corresponds to the silver plating. The silver plating was covered with a mixture of corrosion products and sediments. The corrosion products above the silver plating were mainly composed of copper carbonates. During the burial period, a layer of corrosion products containing mainly copper oxides had developed below the silver plating. The treatment selected was an application of disodium EDTA gels at different concentrations (0.5, 2 or 5 wt%). It was applied to one half of the surface of a coin for 20 min, four or five times. The coins had not undergone mechanical cleaning before the first application of the gel. The treated surface was rinsed using a cotton swab impregnated with acetone diluted to 50% v. The coins were photographed prior to treatment and after each application of gel. The results achieved after the final application are presented in Figure 3. All treatments with 0.5 wt% di-EDTA were ineffective. Gel treatment with 2 wt% di-EDTA partially removed corrosion products but was not complete after five applications. Gel treatment with 5 wt% di-EDTA ensured effective cleaning after four or five applications. Peelable hydrogels (agar gels or gellan gum) gave the best results: they ensured good contact between the gel and the surface of the object; the treatment was effective when the solution contained a sufficient concentration of di-EDTA. They were easily removed and left little visible residue on the surface of the coin; post-treatment rinsing was therefore limited and the result of the treatment was homogeneous. The application of these hydrogels is precise and allows for the treatment of a well-defined surface. Viscous hydrogels (xanthan gum) also effectively cleaned the surface, but after treatment the surface was less well defined and gel removal was difficult. Xanthan gum left a large amount of residue that required copious rinsing. Despite thorough rinsing, it is possible that residue may have penetrated into the cracks and remains on the surface of the archaeological object. 

#### 2.2.4. Cleaning of Lead Artefacts

**Figure 4:**
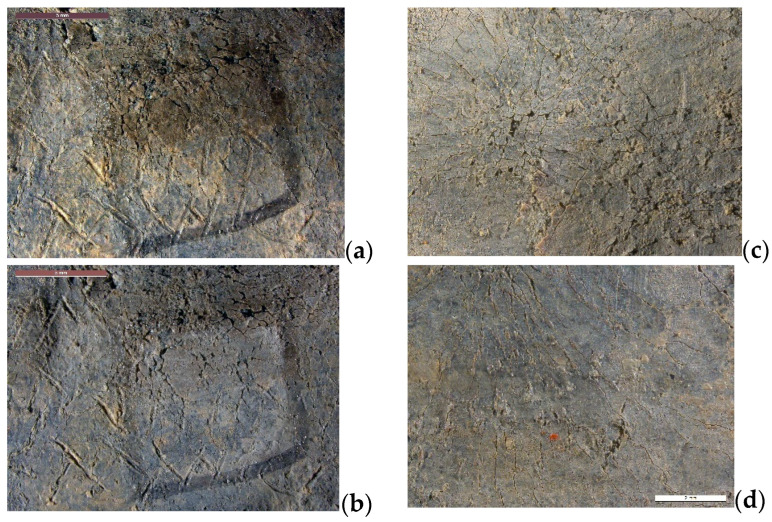
Results of gel treatments on curse tablet: before (**a**) and after application of agar with oxalic acid (**b**), before (**c**) and after application of xanthan with citric acid (**d**), (© L. Rossetti/Arc’Antique—Grand Patrimoine de Loire Atlantique, Nantes, France).

The fourth case study concerns another archaeological object: a curse tablet from the collection of the Medals and Antiques Department of the National Library of France. Most of the tablets from this collection come from North Africa, date from the early centuries AD, and were acquired in the 19th century. Such tablets were used as magical objects and some of them are inscribed [31]. Their condition had degraded as a result of being conserved in oak coin cabinets and exposed to acetic acid vapors. The inscriptions were either completely covered over by hard, thick corrosion products, or lightly veiled with whitish corrosion products. Layers containing the incisions sometimes either lacked adherence and displayed extensive cracking or, inversely, were compact and solid. The cleaning objective was to remove corrosion products so that the inscriptions could be read. With regard to chemical treatments, tests by conservators revealed that only acidic solutions could eliminate corrosion products from lead. Peelable hydrogels were not very compatible with acidic solutions. A film of water agar was first formed on the tablet and took on the shape of the surface. It was then immersed for 2 h in an oxalic acid solution. Next, it was placed on the relevant surface for 2 h. The operation was repeated three times. The photo in Figure 3b shows that the gel shrank after immersion in the acidic solution, and although it retained its mechanical strength, it was degraded. The contact between the gel film and the surface to be treated was not as good as when the agar gel was applied hot. The cleaning resulting from the use of oxalic acid in agar gel was not completely successful (Figure 4): The layer of corrosion products was reduced but only in the areas in direct contact with the gel. In addition, a whitish veil formed on the surface and increased with the number of gel applications. Xanthan gels were more compatible with acidic solutions. The cleaning test was performed with citric acid (5 wt%) in xanthan gel. The gel was applied for 1 h and the operation was repeated twice. The surface was rinsed using a cotton swab impregnated with water, but the gel proved very difficult to remove completely. Figure 4 shows the detail of the cleaned surface: corrosion products are still largely present and the treatment was not really effective. Gel treatments are therefore not really suitable for this type of archaeological object. Mechanical cleaning (micro-sandblasting with plant-based abrasive or with cationic exchange resins) yielded better results.

#### 2.2.5. Cleaning of Painted Aluminum Alloys

**Figure 5:**
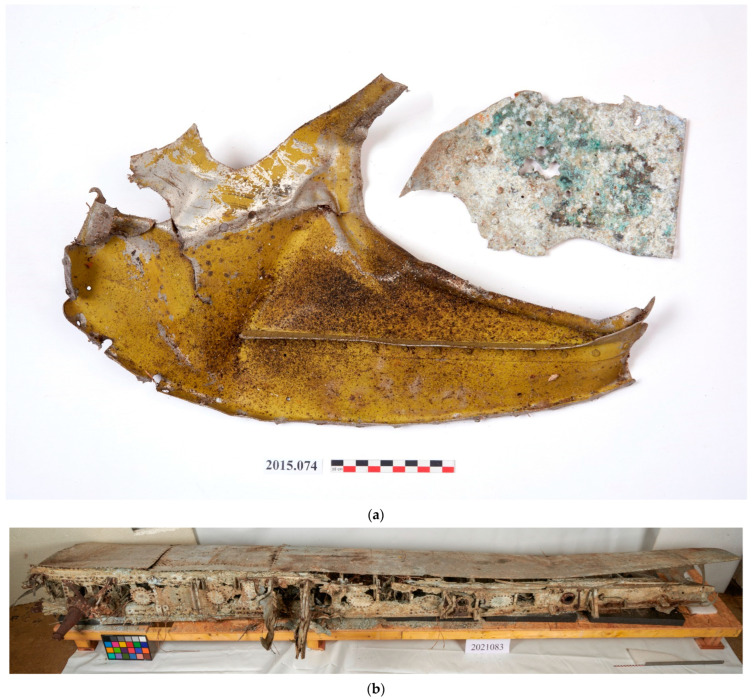
A small plate fragment from a Messerschmitt Bf109 (**a**) and a wing part from a Spitfire Mk VII MB887 (**b**) (© L. Preud’homme/Arc’Antique—Grand Patrimoine de Loire Atlantique, Nantes, France).

In this case study, cleaning tests were carried out on painted surfaces from two authentic WWII aircraft wrecks:

A plate fragment from a Messerschmitt Bf109, which crashed during WWII at Le Rheu (west of Rennes, France) (Figure 5a). The fragment is in a good state of conservation. It is a copper-based aluminum alloy with cladding (Alclad: layer of pure aluminum above the alloy), it has a layer of paint with good cohesion with the metal surface. The object’s surface was covered with black deposits. The cleaning objective was to remove these black deposits without damaging the paint.

A wing part from a Spitfire Mk VII MB887, which crashed on 1st June 1944, off the Saint Brieuc coast (France) (Figure 5b). The wing part is in relatively good condition: It is also a copper-based aluminum alloy with cladding coated with remains of the original paint. On the object’s surface, there were aluminum corrosion products, some concretions and some traces of iron corrosion products. The cleaning objective was to remove the corrosion products and concretions without damaging the paint remains.

**Figure 6:**
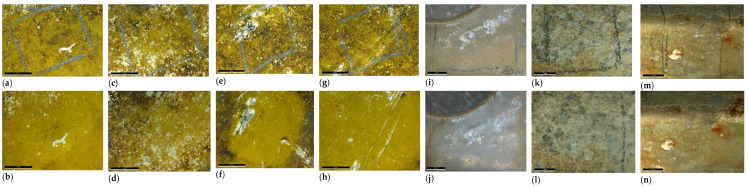
Results of gel treatments on the fragment from a Messerschmitt Bf109: before (**a**) and after treatment using agar with citric acid (0.055 M) (**b**), before (**c**) and after treatment using agar with TAC (**d**), before (**e**) and after treatment using xanthan with metasilicate sodium (**f**), before (**g**) and after treatment using xanthan with tetra-EDTA (**h**) (© S. Bampitzaris/Arc’Antique—Grand Patrimoine de Loire Atlantique, Nantes, France). Results of gel treatments on the wing part from a Spitfire Mk VII MB887: before (**i**) and after treatment using agar with TAC (**j**), before (**k**) and after treatment using xanthan with metasilicate sodium (**l**), before (**m**) and after treatment using xanthan with tetra-EDTA (**n**) (© E. Paillaux/Arc’Antique—Grand Patrimoine de Loire Atlantique, Nantes, France).

For Al alloys, chemical cleaning protocols are still experimental and limited data are available. The main treatments were developed in Australia and use solutions of citric acid [32], sodium metasilicates or tetrasodium EDTA [33]. Tri-ammonium citrate (TAC) solutions were also tested as they are used on painted surfaces [34]. For sodium metasilicate and tri-sodium EDTA solutions, xanthan gels were applied, agar gels were used for citric acid (0.055 M) and TAC. The results for the Messerschmitt Bf109 fragment are shown in Figure 5. The best results were obtained with citric acid or sodium metasilicate solutions: black deposits were removed with no damage to the paint. The ammonium citrate solution removed most of the black deposits, but it also eliminated the thinner parts of the painted layer. The EDTA solution was effective: all the black deposits disappeared but the paint was damaged by the cleaning. Different treatment solutions were also tested on the Spitfire wing part (Figure 6). Metasilicate sodium had little effect on corrosion products even after 60 min of treatment: this solution was therefore not suitable for cleaning the Spitfire wing. TAC or EDTA were more effective solutions because of their action on corrosion products. When tetra- and disodium EDTA solutions were applied using gel, they partially removed the corrosion products and had a slight effect on concretions but they also caused the paint remains to deteriorate. The TAC gel solution offered the best compromise for removing corrosion products without damaging the traces of paintwork but did not completely clean the object. To clean the wing part, agar gel with TAC was applied with a spray gun, in line with accepted procedures for large surfaces [21]. Three 20 min applications were necessary for a satisfactory result. Dirt was removed and rust stains reduced. Then, to homogenize the surface of those parts with a larger quantity of corrosion products and concretions, they were cleaned by sandblasting with vegetal abrasive.

#### 2.2.6. Cleaning of Vermeil Gold

**Figure 7:**
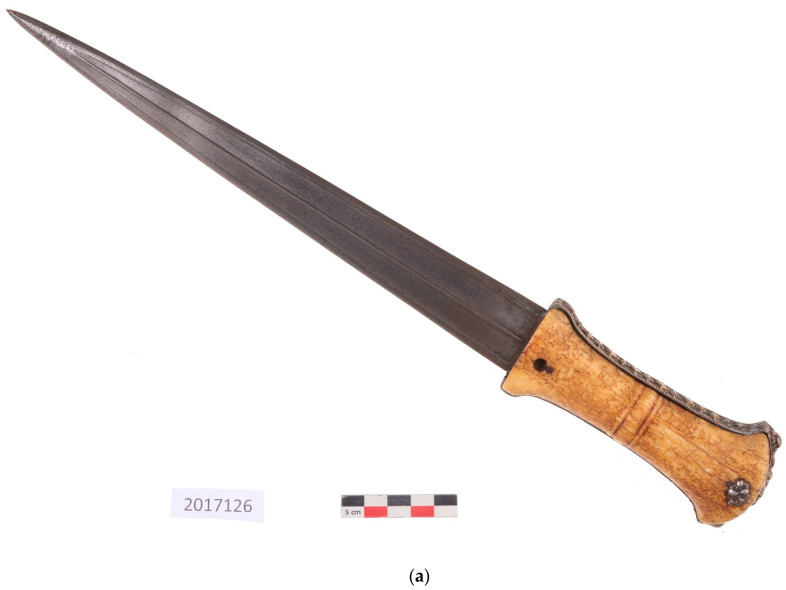

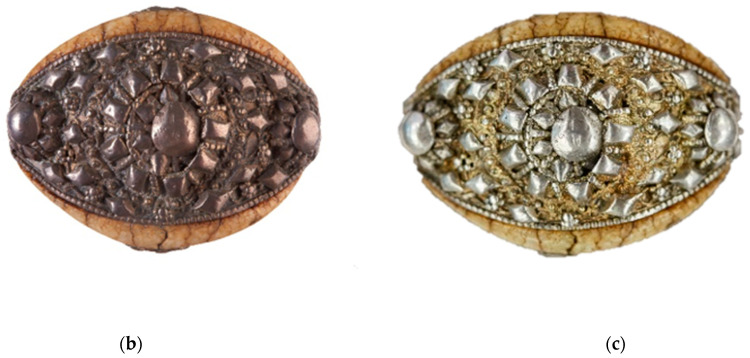
A knife from the collection of the Pierre Loti Museum (**a**), and of a detail of the handle before (**b**) and after treatment (**c**) (© J.G. Aubert/Arc’Antique—Grand Patrimoine de Loire Atlantique, Nantes, France).

This new case study concerns the cleaning of a further object from the Islamic art collection of the French author, Pierre Loti. It is a dagger in a good state of conservation (Figure 7a), composed of a steel blade and a walrus ivory handle with silver and vermeil plating. The silver elements displayed fine, homogeneous silver sulfide corrosion (Figure 7b). Tarnished silver objects are often cleaned mechanically but the relief of the decoration made it difficult to apply a mechanical treatment. Electrochemical treatments thus provided a good alternative [35]: First, the silver sulfides were reduced to silver at −0.89 V/SCE (saturated calomel electrode), then the reduced silver deposited on the gilding was oxidized at +0.66 V/SCE to reveal the gilding. However, since the dagger is a composite object, the ivory parts could not be immersed. Electrochemical treatment was therefore applied locally using an agar gel with KNO_3_ at 1 wt%. Cleaning was completed by rinsing the surface with a water/ethanol mixture applied with a soft brush (Figure 7c). In this case study, the gel treatment was successful.

#### 2.2.7. Stabilization of Copper Alloys or Iron

**Figure 8:**
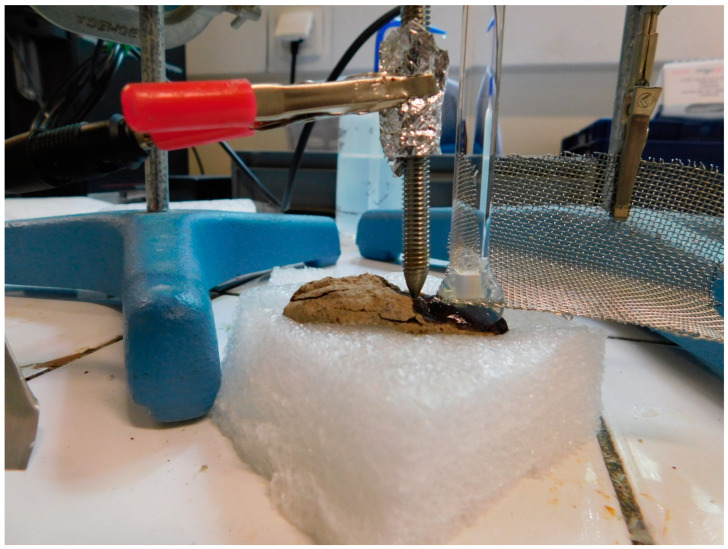
The electrolytic assembly for the stabilization using gel of an archaeological iron object (© C. Fontaine/Arc’Antique—Grand Patrimoine de Loire Atlantique, Nantes, France).

As seen in the previous case, local electrochemical treatments offer a very satisfactory treatment alternative for composite objects. Other electrochemical treatments have been developed for the treatment of metals, in particular dechlorination treatments for iron [36] or copper alloys [37]. The electrochemical setup is a classic three-electrode electrolysis setup. The object to be treated acts as the working electrode. The gel (agar with KNO_3_ 1 wt%) acts as an electrolytic solution and the counter electrode is a stainless-steel grid. The reference electrode is placed in an extension containing a conductive solution (KNO_3_ 1 wt%) and inserted in the gel (Figure 8). The reference electrode is a saturated calomel electrode (SCE). Cathodic potential is applied to the object to be treated (working electrode) to allow chloride ions to migrate into the gel: the potential is −1.04 V/SCE for iron objects or −0.19 V/SCE for copper alloys. A previous study has shown the effectiveness of this treatment [38]. For it to be effective, contact between the corrosion layers and the gel must be very good; a drop of the electrolyte (KNO_3_ at 1 wt%) must be placed on the surface to be treated. The gel must be changed every 30 min. The difficulty of these electrochemical treatments using gel is to determine the end of treatment. The amount of extracted chloride can be monitored by measuring chloride concentration in the gel after treatment by X-ray fluorescence (XRF) [38]. When all chlorides have been extracted, the object is stable. The absence of active corrosion can also be checked by measuring the oxygen consumption of the object in a leakproof pocket [39].

## 3. Discussion

**Figure 9:**
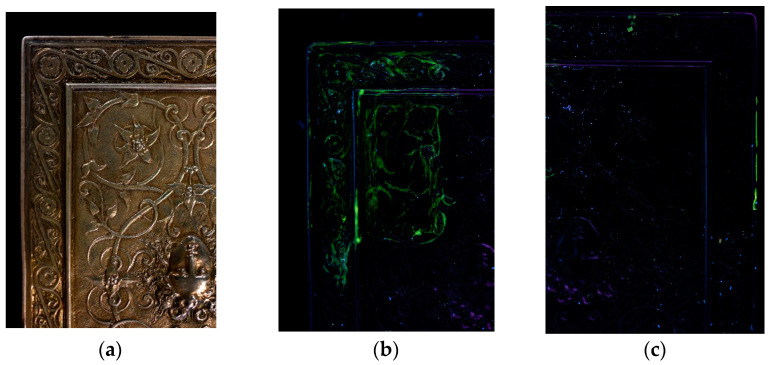
A historical silver object with surface relief: (**a**) under normal lighting, (**b**) under UV light after removal of a xanthan gel containing 10^−4^ M fluorescein, (**c**) under UV light after removal of an agar gel containing 10^−4^ M fluorescein (© E. Guibert-Martin/Arc’Antique—Grand Patrimoine de Loire Atlantique, Nantes, France).

These first studies on the use of gels for the treatment of metals show that gels can provide good treatment alternatives but that they are not suitable in every case. Treatments using agar gels applied hot yielded the best results. The limits of hot applied agar gels are their compatibility with treatment solutions. Not all active agents commonly used in chemical treatments are compatible with agar. The treatment solutions must have a pH close to the neutral range, otherwise the helix network of the agar gel cannot form and the gel is not peelable. Agar gel treatments can incorporate solutions of TAC, EDTA, dilute citric acid or a neutral conductive solution such as KNO_3_. The case studies showed that gel treatments work better on historical than archaeological objects. This is due to difference in the surface state of the objects. Generally, historical objects have a homogeneous surface whereas archaeological objects have cracked, heterogeneous corrosion layers. Agar gels applied hot to an archaeological object tend to penetrate the cracks and porosities of the corrosion layers. When the gel is removed, parts of the surface may become detached and residues may remain in the corrosion layers. Post-treatment gel residue has not yet been quantified. An initial study evaluated the presence of residues by integrating a fluorescent marker, fluorescein (at 10^−4^ M), in the gels. The technique was not precise enough to determine the quantity of the residues, but it enabled a comparison to be made with residues due to agar or xanthan gels, respectively, on a historical silver object with a surface relief (Figure 9). After removal of the gel, the surface was rinsed with demineralized water using a cotton swab. The green color of the gel residues showed up under ultraviolet light (UV). Agar residues were very localized in cavities and represented about 0.7% of the treated surface while xanthan residues formed a veil on the surface and represented about 20% of the treated surface. Although xanthan gels are more compatible with chemical agents than agar gels, they form a viscous gel that is difficult to rinse off. Xanthan residues are significant and are the main reason that conservators avoid using it for the treatment of metal objects.

Gellan gels and agar gels form peelable gels and have similar compatibility with chemical agents. The advantage of agar gels is their thermo-reversibility: they can be reheated several times, which is not the case for gellan gels. Moreover, the low cost of agar gels makes them easy for conservators to access. Gellan gels solidify very quickly because they have a gelling temperature of 70 °C while agar gels gel at around 40 °C. Agar gels may seem more practical to use but in hot weather they may become unusable and unable to solidify. In this case, the use of gellan gels is to be preferred.

Another advantage of using gels is the possibility of localized treatment. They are particularly suited to the treatment of composite objects that cannot withstand immersion in a chemical bath. The use of gel also allows the quantity of solution and active agent to be limited. This is often cited as an ecological benefit of gel treatments. Gel treatment as a new green method is currently being researched by Edith Joseph at the University of Neuchatel [8]. The use of siderophores for the cleaning of historical ferrous metals yields very promising results [40].

These different studies are shared among French conservators who are participating in the “Gels Métaux” collaborative project [41]. Through a series of exchange days and workshops, scientists and conservators present their respective studies and treatment examples as well as the success and limits of their research.

## 4. Conclusions and Perspectives

For several decades, the use of gels has greatly modified the practices of painting and graphic art conservators. Progressively, they are now becoming more commonplace in the restoration of metals. The use of cleaning gels for historical objects is increasingly becoming an integral part of conservation practice. The best results were obtained using agar gels associated with a chelating agent (EDTA or TAC) applied hot. These gel treatments were used to remove dirt and corrosion products from historical copper and iron objects. They are particularly suitable for composite objects. For objects featuring decoration (gilding, silver plating, painting), gel treatments can be an interesting alternative. Gel treatments allow for the local application of a chemical or electrochemical treatment. Peelable gels are preferable on surfaces in good condition, such as those of historical objects. The hot application of these peelable gels (agar or gellan gum) ensures good contact between the gel and the surface to be treated. The use of gel films is generally unsuitable for the treatment of metal objects because the geometry of the objects prevents good contact between the gel film and the treated surface. Viscous gels, such as xanthan gum, adhere well to the surface of the object and have a good level of compatibility with many active agents. However, xanthan gels are very difficult to remove, even after copious rinsing. On a surface in good condition—as is generally the case for historical objects—even after thorough rinsing a large quantity of xanthan gel residue remains in the form of a thin layer spread over the surface.

For archaeological objects, the use of gels is more problematic. Gels can penetrate corrosion layers and cause peeling or/and leave residues after treatment. The question of residues is an issue that remains to be investigated. Gel residues must be quantified and their impact on the conservation of objects must be determined. It should also be possible to limit their presence by optimizing gel application protocols.

## 5. Material and Methods

### 5.1. Preparation Protocol of Gels

To prepare agar gel, first a solution was heated to 50 °C and mixed with 3 wt% agar (AgarArt^®^ provided by CTS Conservation, France). It was then heated to 90 °C until the mixture became homogeneous and translucent. It was allowed to cool for 24 h before being heated a second time to 90 °C. The preparation of gellan gum (Kelcogel^®^ provided by CTS or Phytagel^®^ from Sigma Aldrich), requires only a single heating. When peeling gels (agar or gellan gum) was applied hot, the gel was mixed directly with the treatment solution and applied to the object with a syringe (hot application). For cold application, films (2 or 3 mm in thickness) were formed with demineralized water. The films were then immersed in the treatment solution for 2 h at room temperature, impregnating the gel with the treatment solution.

Xanthan gum preparations (from Kremer, or Vanzan^®^ by CTS Conservation) were made at room temperature 24 h prior to use. A concentration of 2 wt% (Kremer) or 5 wt% (CTS Conservation) xanthan gum was added to the treatment solution, and after 24 h the mixture became homogeneous. The gel can be applied cold. Unused gel must be refrigerated (24 h) to avoid the development of mold.

### 5.2. Treatment Solutions

The active agents selected are used in chemical or electrochemical treatments in metal restoration. The 4 types of selected active agents tested were: acidic solutions to eliminate corrosion products, sediments and concretions; neutral or alkaline conductive solutions to carry out electrolysis, and chelating agent solutions to remove corrosion products. The list of selected active agents is detailed in Table 2.

## Figures and Tables

**Table 1:** Compatibility of treatment solutions with physical polysaccharide-based hydrogels (agar, gellan gum and xanthan gum).

Hydrogels	Treatment Solutions	Compatibility
Agar	Nitric acid (10^−4^ M)	Possible with pre-made film
	Citric acid (5 wt%)	Possible with pre-made film
	Citric acid (0.055 M)	Compatible (hot application)
	Oxalic acid (5 wt%)	Possible with pre-made film
	KNO_3_ (1 wt%)	Compatible (hot application)
	TAC (2.5 or 5 wt%)	Compatible (hot application)
	di-EDTA (0.5–2.5 or 5 wt%)	Compatible (hot application)
	tetra-EDTA (5 wt%)	Compatible (hot application)
	Metasilicate sodium (2 wt%)	Incompatible
	NaOH (2 wt%)	Incompatible
Gellan gum	Nitric acid (10^−4^ M)	Incompatible
	Citric acid (5 wt%)	Incompatible
	Citric acid (0.055 M)	Compatible (hot application)
	Oxalic acid (5 wt%)	Incompatible
	KNO_3_ (1 wt%)	Compatible (hot application)
	TAC (2.5 or 5 wt%)	Compatible (hot application)
	di-EDTA (0.5–2.5 or 5 wt%)	Compatible (hot application)
	tetra-EDTA (5 wt%)	Compatible (hot application)
	Metasilicate sodium (2 wt%)	Incompatible
	NaOH (2 wt%)	Incompatible
Xanthan gum	Nitric acid (10^−4^ M)	Possible but very viscous
	Citric acid (5 wt%)	Possible but very viscous
	Citric acid (0.055 M)	Compatible
	Oxalic acid (5 wt%)	Possible but very viscous
	KNO_3_ (1 wt%)	Compatible
	TAC (2.5 or 5 wt%)	Compatible
	di-EDTA (0.5–2.5 or 5 wt%)	Compatible
	tetra-EDTA (5 wt%)	Compatible
	Metasilicate sodium (2 wt%)	Possible but very viscous
	NaOH (2 wt%)	Incompatible

**Table 2:** List of treatment solutions.

Type	Composition	Concentration	pH
Acidic solution	Nitric acid	10^−4^ M	3
	Citric acid	5 wt%	3
	Citric acid	0.055M	5.4
	Oxalic acid	5 wt%	2
Neutral conductive solution	KNO_3_	1 wt%	5.6
Alkaline solution	Metasilicate Sodium	2 wt%	9
	NaOH	2 wt%	13.5
Chelating agent	TAC (tri-ammonium citrate)	2.5 wt%	7
	TAC (tri-ammonium citrate)	5 wt%	7.5
	Disodium EDTA (ethylenediaminetetraacetic acid)	0.5 wt%	4.7
	Disodium EDTA (ethylenediaminetetraacetic acid)	2 wt%	4.5
	Disodium EDTA (ethylenediaminetetraacetic acid)	5 wt%	4.4
	Tetrasodium EDTA (ethylenediaminetetraacetic acid)	5 wt%	10

## Data Availability

The data of these studies are kept only at the Arc’Antique laboratory.
